# Significance of RCC2, Rac1 and p53 Expression in Breast Infiltrating Ductal Carcinoma; An Immunohistochemical Study

**DOI:** 10.30699/IJP.2024.2014367.3198

**Published:** 2023-02-15

**Authors:** Aiat Shaban Hemida, Reham Ahmed Abdelaziz, Moshira Mohammed Abd El-Wahed, Nancy Yousef Asaad, Marwa Mohammed Serag El-Dien, Hend Ali Elshahat Ali

**Affiliations:** 1 *Department of Pathology, Faculty of Medicine, Menoufia University, Shebin El Kom, Egypt*; 2 *Department of Clinical Oncology& Nuclear Medicine, Faculty of Medicine, Menoufia University, Shebin El Kom, Egypt*

**Keywords:** Breast neoplasms, Cell cycle proteins, Rac1 GTP-binding protein, Tumor suppressor protein p53

## Abstract

**Background & Objective:**

The regulator of chromosome condensation 2 (RCC2) and RAS-related C3 botulinum toxin substrate 1 (Rac1) have been implicated in the promotion of breast cancer cell proliferation and migration. The signaling pathway involving p53/RCC2/Rac1 has been proposed to contribute to the regulation of colon cancer metastasis. However, until now, this pathway has not been thoroughly investigated in breast cancer. This study seeks to explore the influence of immunohistochemical expression and the correlation among RCC2, Rac1, and p53 in breast infiltrating ductal carcinoma (IDC).

**Methods:**

Immunostaining was performed on 120 breast IDC specimens using RCC2, Rac1, and p53 antibodies. Statistical analyses were conducted to examine the correlations between these antibodies.

**Results:**

A Positive expression of RCC2, Rac1, and p53 was observed in 116 (96.7%), 120 (100%), and 33 (27.5%) of the breast cancer cases, respectively. RCC2, Rac1, and p53 demonstrated association with poor prognostic parameters such as frequent mitoses, high Ki-67 status, positive lymphovascular invasion (LVI), and advanced tumor stage. A highly significant direct correlation was found between each immunohistochemical marker and the other two markers. Shorter overall survival was linked to multifocal tumors (*P*=0.017), advanced tumor stage (T3) (*P*=0.010), Luminal B subtype (*P*=0.015), progressive disease (*P*=0.003), positive Her2neu status (*P*=0.008), and metastasis to distant organs (*P*<0.001). However, RCC2, Rac1, and p53 did not exhibit a significant association with overall survival.

**Conclusion:**

The high expression levels of RCC2, Rac1, and p53 in breast IDC suggest their potential role in tumor behavior. The association of RCC2 and Rac1 with poor prognostic parameters may serve as predictive indicators for aggressive tumors, thus implying that targeted therapy could be beneficial in the treatment of breast cancer.

## Introduction

Globally, breast cancer ranks as the most frequently diagnosed cancer in females and stands as the second leading cause of cancer-related mortality among women (1). In Egypt, breast cancer represents the predominant malignancy in women, comprising 32.4% of all cancers in this demographic (2). Infiltrating duct carcinoma (IDC) emerges as the prevailing form of breast cancer (3). 

The regulator of chromosome condensation 2 (RCC2) has been implicated in breast cancer by fostering cell proliferation and migration (4). RAS-related C3 botulinum toxin substrate 1 (Rac1) is involved in regulating various aspects of the tumor cell cycle, apoptosis, proliferation, invasion, migration, and angiogenesis (5). Furthermore, it has been suggested to modulate tumor stem cells and facilitate tumor genesis (6), potentially influencing drug resistance and prognosis in cancer patients (7).

The p53 protein is known to regulate critical cellular processes such as the cell cycle, DNA repair, and apoptosis. It governs genes implicated in metastatic pathways, including cell adhesion, motility, and invasion (8). In normal cells, p53 exhibits transient expression and a very short half-life. However, mutation in the p53 gene results in protein stabilization within the cell nucleus, compromising its growth-suppressing function and fostering cellular proliferation and anti-apoptotic activities, particularly during cellular quiescence (9). 

A previous study on colon cancer revealed that p53 activates RCC2 transcription by binding to its promoter. RCC2 deficiency induces alterations in cell morphology and activates Rac1, promoting cell migration. This underscores the potential involvement of the p53/RCC2/Rac1 signaling pathway in colon cancer metastasis regulation (10). Nevertheless, the correlation among p53/RCC2/Rac1 proteins in breast carcinoma remains unexplored. Thus, the objective of this study is to examine the immunohistochemical expression and correlation of RCC2, Rac1, and P53 in breast IDC and investigate their prognostic implications and associations with various clinicopathological parameters.

## Material and Methods

This retrospective study comprised 120 specimens of breast infiltrating ductal carcinoma (IDC). Paraffin blocks were obtained from the archival material of the Department of Pathology at the Faculty of Medicine, Menoufia University Hospital, covering the period from January 2018 to December 2020. All selected cases represented IDC that had undergone either mastectomy or conservative breast surgery. Cases subjected to neo-adjuvant therapy were excluded from the study. 

**Ethical Considerations**

The study received approval from the institutional ethics committee in compliance with the Helsinki Declaration of 1975 (revised in 2000) (IRB: 7/2020 PATH17). 

**Clinicopathological Evaluation**

Data were gathered from patients' files and through the re-evaluation of Hematoxylin and Eosin (H&E) stained sections. Grading followed the Elston/Nottingham modification of the Bloom/Richardson system (11, 12). Staging was determined according to the TNM staging system, based on the 8^th^ edition of the American Joint Committee on Cancer (AJCC) (13). The Nottingham prognostic index (NPI) was calculated (14).

Using low power magnification (100x), the amount of stromal tumor-infiltrating lymphocytes (TILs) was categorized as low, intermediate, or high (15). Lymphovascular invasion was assessed (16). Mitotic and apoptotic figures were tallied in ten randomly selected high-power fields (HPFs) (400x), corresponding to at least 1000 malignant cells (17, 18). Molecular classification was performed following staining of all cases with ER, PR, Her2neu, and Ki-67 (19-22).

**Tissue Microarray Blocks**

All mastectomy cases included in the study were utilized to construct 14 duplicate-core tissue microarray (TMA) blocks using a manual tissue microarrayer (Breecher instrument Manual Microarray, Wisconsin, USA). 

**Immunohistochemistry**

The immunostaining technique employed utilized the streptavidin-biotin amplified system. The primary antibodies included ready-to-use IgG Rabbit monoclonal anti-RCC2 antibody (Chongqing Biospes Co., LTD, China, Catalog Number: YPA1680), anti-Rac1 antibody (Chongqing Biospes Co., LTD, China, Catalog Number: YPA2224), and anti-p53 antibody (Biocare Medical, USA, Catalog Number: IP 298 G10). Antigen retrieval was conducted by boiling the tissue sections in EDTA for all three antibodies. Human spleen, human prostatic tissue, and p53-positive breast cancer tissues were employed as positive control tissues for RCC2, Rac1, and p53, respectively. Sections stained without the primary antibody were used as negative controls.

**Immunohistochemical Staining Interpretation**

Positive expression of RCC2, Rac1, and p53 was identified through the presence of brownish nuclear staining (for RCC2 and p53) and cytoplasmic ± nuclear staining for Rac1 (23-25).

The percentage of stained invasive tumor cells was estimated, and staining intensity in tumor epithelial cells was categorized as mild, moderate, or strong. The H-Score was calculated using the formula:

H-Score = (1 × % of mildly stained cells) + (2 × % of moderately stained cells) + (3 × % of strongly stained cells) (23, 26).

**Response to Treatment and Survival**

Patients received treatment and follow-up care at the Clinical Oncology and Nuclear Medicine department, Faculty of Medicine, Menoufia University. Data collected from patient files included family history, comorbidities, metastatic sites, response to treatment, progression-free survival, and overall survival. Response to treatment was evaluated following the RECIST guideline, version 1.1 (27). 

**Statistical Analysis**

The collected data underwent analysis using a personal computer equipped with the Statistical Package for the Social Sciences (SPSS) version 22 (SPSS Inc., Chicago, USA). Qualitative data were presented in numbers and percentages, while quantitative data were characterized using the range, median, interquartile range (IQR), mean, and standard deviation (SD). Employed statistical tests included the chi-square (χ^2^), Fisher's exact, Student's t-test, ANOVA, Mann-Whitney U, Kruskal-Wallis, McNemar, and marginal homogeneity test. Pearson's correlation coefficient was utilized to gauge the linear correlation between two continuous variables. 

Progression-free survival (*P*FS) was calculated in months from the date of diagnosis to the time of disease progression (either local recurrence or distant metastasis). Overall survival (OS) was determined in months from the date of diagnosis to the last follow-up visit or death. Kaplan-Meier survival curves and Hazard function curves and their 95% confidence interval (CI) were constructed for survival analysis. A P-value less than 0.05 and 0.01 were considered statistically significant and highly significant, respectively (28).

## Results

**Clinicopathological Data of the Studied Cases**

The clinical and pathologic data of studied cases are shown in Table 1

**Table 1:** Clinicopathological data of the studied IDC cases (n = 120)

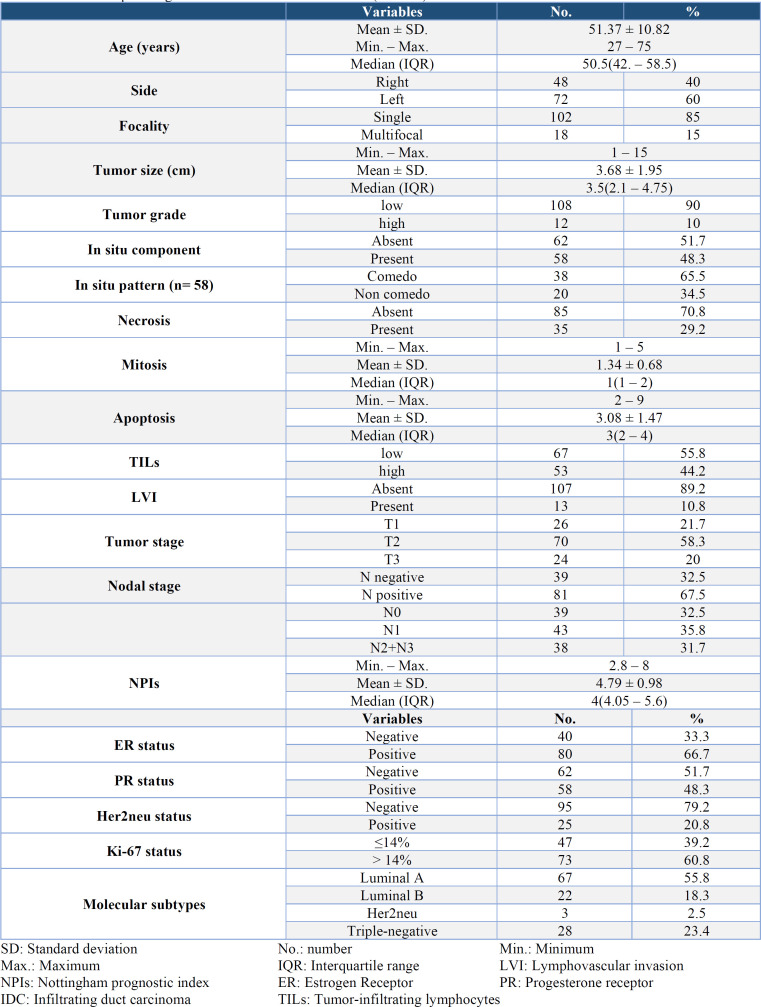

For statistical analysis, grades were categorized into low (grades 1 & and 2) and high (grade 3). Tumor-infiltrating lymphocytes (TILs) were grouped into low TILs (low and intermediate) and high TILs (high group). The Her2neu enriched subtype was excluded from statistical comparisons due to this group's small number of cases.

**Expression of RCC2, Rac1 and P53 in the Studied Cases**

**Fig. 1:**
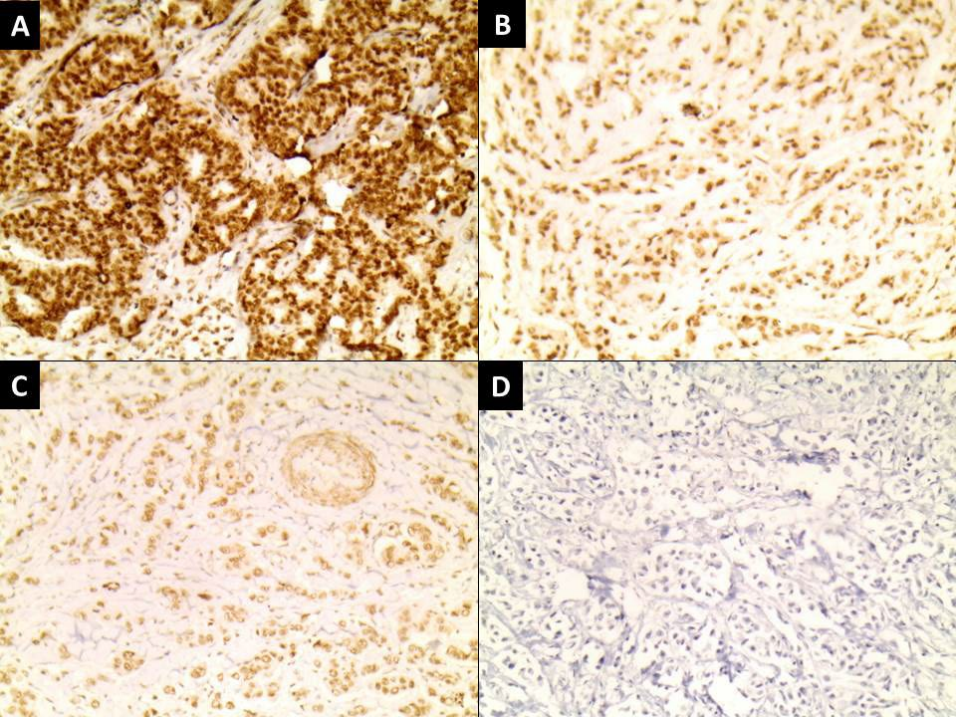
Infiltrating ductal carcinoma showing (A) strong nuclear expression of RCC2 (IHC x200), (B) moderate nuclear expression of RCC2 (IHC x200), (C) mild nuclear expression of RCC2 (IHC x200), (D) negative expression of RCC2 (IHC x200).

**Fig. 2:**
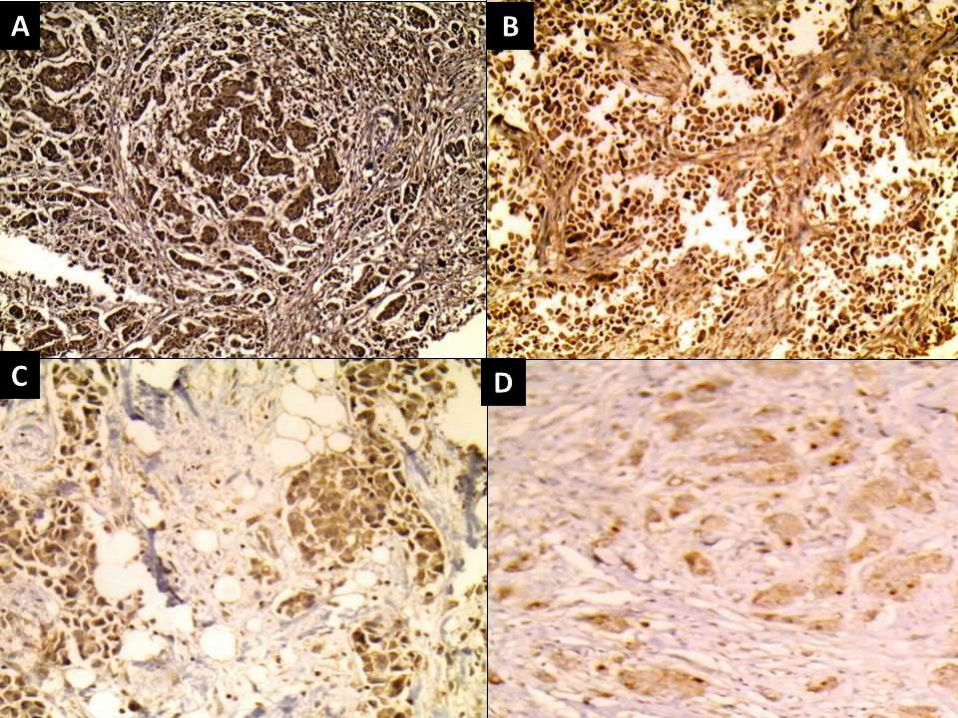
Infiltrating ductal carcinoma showing (A) strong nuclear ± cytoplasmic expression of Rac1 (IHC x40), (B) strong nuclear ± cytoplasmic expression of Rac1 (IHC x100), (C) moderate nuclear ± cytoplasmic expression of Rac1 (IHC x200), (D) mild nuclear ± cytoplasmic expression of Rac1 (IHC x200).

**Fig. 3:**
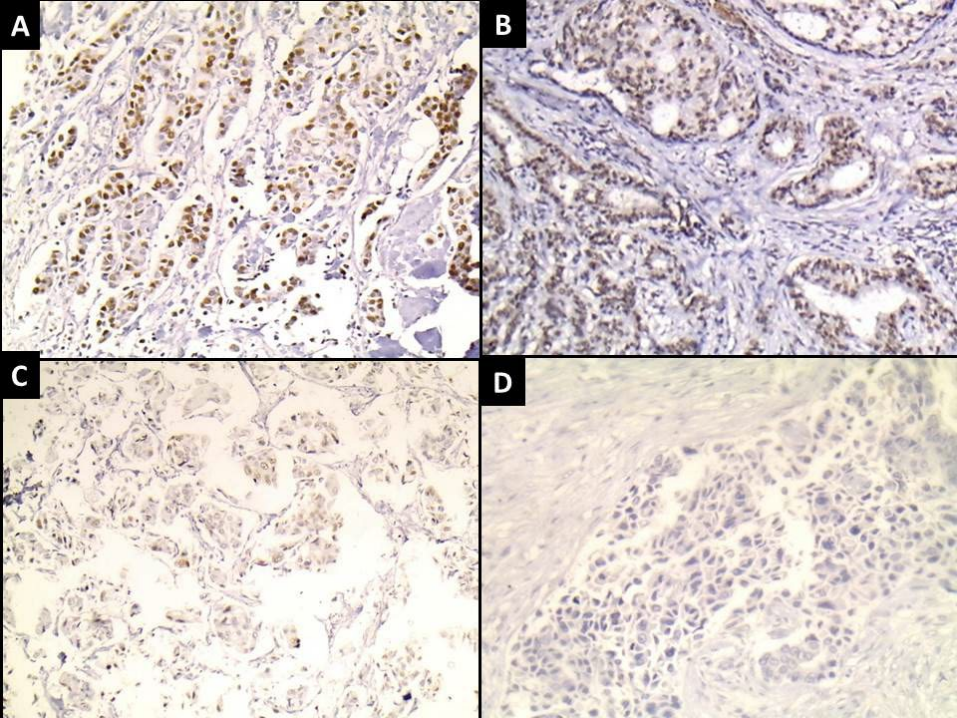
Infiltrating ductal carcinoma showing (A) strong nuclear expression of P53 (IHC x100), (B) moderate nuclear expression of P53 (IHC x100), (C) mild nuclear expression of P53 (IHC x100), (D) negative expression of P53 (IHC x200).

The results of immunostaining for RCC2, Rac1, and p53, including expression (negative/positive), percentage, and intensity, are presented in Table 2 (Figures 1, 2, 3).

**Table 2:** Expression of RCC2, Rac1 and P53 in the studied IDC cases

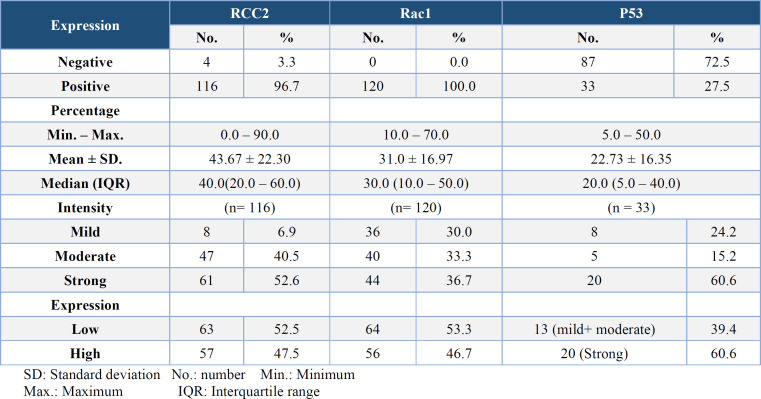

Given the widespread expression of RCC2 in nearly all studied cases (116/120), the cases were stratified into low and high-expression groups based on the median of the H-score expression. 

The studied cases were categorized into low and high Rac1 expression groups according to the median of the H-score expression. Regarding p53 expression, the cases were grouped into mild (mild + moderate) versus strong intensity (strong) categories.

**Association of** **RCC2** **Expression with the Clinicopathological Parameters**

A highly significant association was observed between high expression of RCC2 and frequent mitoses (*P*≤ 0.001), a high apoptotic count (*P*≤0.001), and a high Ki-67 status (*P*≤0.001). Additionally, a significant association was noted between the high expression of RCC2 and the presence of LVI (*P*=0.024) (Table 3).

**Fig. 4:**
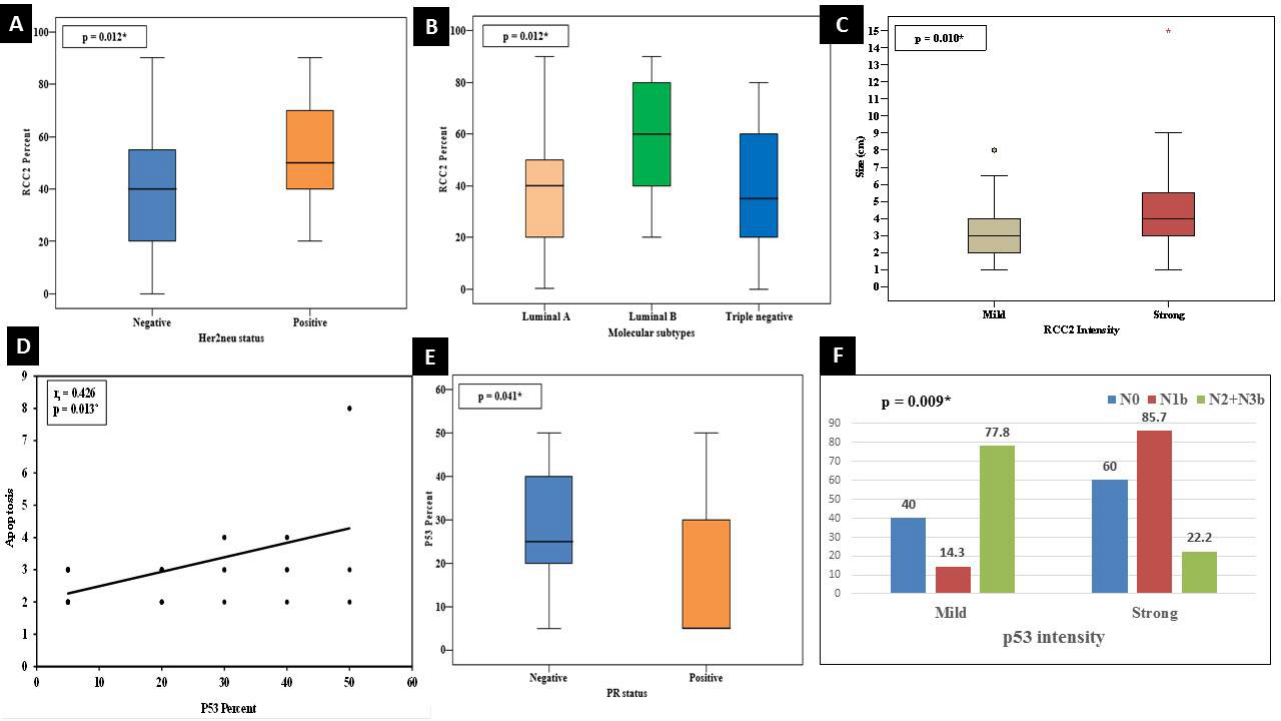
Infiltrating ductal carcinoma with Her2neu positive status (A) and luminal B subtype (B) showing a significantly higher RCC2 expression. Large tumor size (C) shows a significant association with strong RCC2. Positive correlation between p53 percent and apoptosis (D) Significant relation between positive PR and low p53 expression (E) Strong p53 intensity is associated with N0 and N1 nodal stage (F)

Furthermore, a significant association was found between a higher percentage of RCC2 expression and positive Her2neu status (*P*=0.012) (Figure 4A). Molecular subtypes of IDC exhibited a significant association with RCC2 expression percentage (*P*= 0.012), with post hoc testing revealing that luminal B cases were associated with the highest median percentage of RCC2 expression (Figure 4B).

Concerning RCC2 intensity of expression, a significant association was observed between larger tumor size and strong RCC2 intensity of expression (*P*=0.010) (Figure 4C).

**Table 3:** Relation between RCC2 low/high expression and the clinicopathological parameters in all the studied breast IDC cases (n= 120)

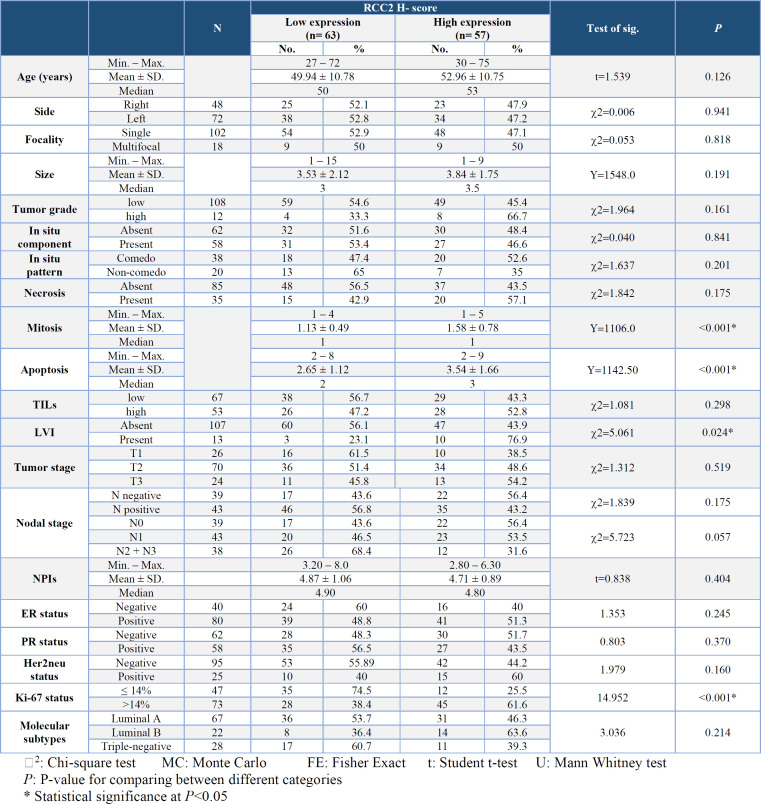

**Association of Rac1 Expression with the Clinicopathological Parameters**

High Rac1 expression demonstrated a highly significant association with a high Ki-67 status (*P*≤0.001) and molecular subtypes (*P*=0.009). Post hoc analysis revealed that the luminal B IDC group was significantly associated with high Rac1 expression. Additionally, a significant association was observed between high expression of Rac1 and frequent apoptosis (*P*=0.019), the presence of LVI (*P*=0.021), positive ER (*P*=0.028), positive PR (*P*=0.030), and positive Her2neu (*P*=0.016) (Table 4).

**Association of P53 Expression with the Clinicopathological Parameters**

A highly significant association was observed between positive p53 expression and large tumor size (*P*=0.009), high TILs (*P = *0.008), and high Ki-67 status (*P=*0.001) (Table 5).

Furthermore, a significant positive linear correlation was identified between p53 percentage and apoptotic count (*P*=0.013). Conversely, a significant inverse association was noted between p53 percentage and PR status (*P*=0.041) (Figure 4D & E). Additionally, Figure 4F illustrates a highly significant association between mild p53 intensity and an advanced nodal stage (N2+N3) (*P*=0.009).

**Relationship Between RCC2, Rac1 and P53**

**Fig. 5:**
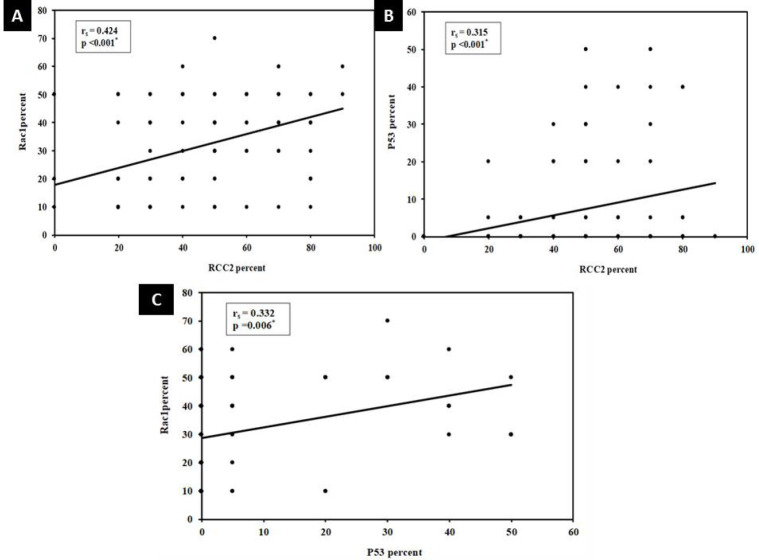
A highly significant direct linear correlation between expression of (A) RCC2 and Rac1 (*P*<0.001, r=0.424), (B) RCC2 and p53 (*P*=<0.001, r=0.315), and (C) Rac1 and p53 (*P*=0.006, r=0.332).

Regarding the association between RCC2, Rac1, and P53 in the studied cases, a highly significant direct correlation was evident between each immunohistochemical marker in this study and the other two markers (Figure 5).

**Table 4:** Relationship between Rac1 as (low/high expression) and the clinicopathological parameters in the studied breast IDC cases (n=120)

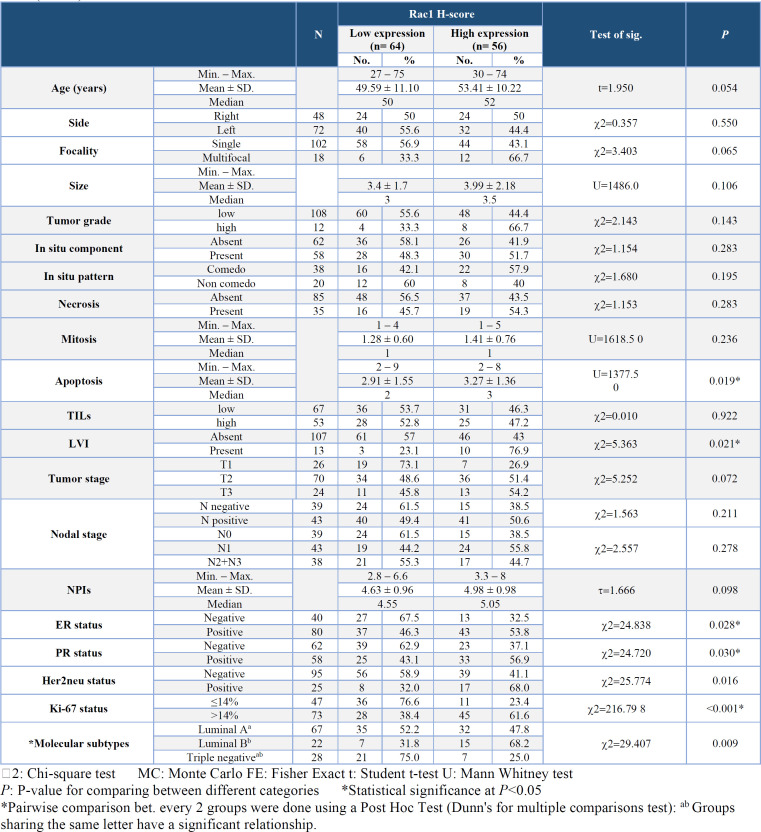

**Table 5:** Relationship between P53 expression and the clinicopathological parameters in all studied breast IDC cases (n= 120)

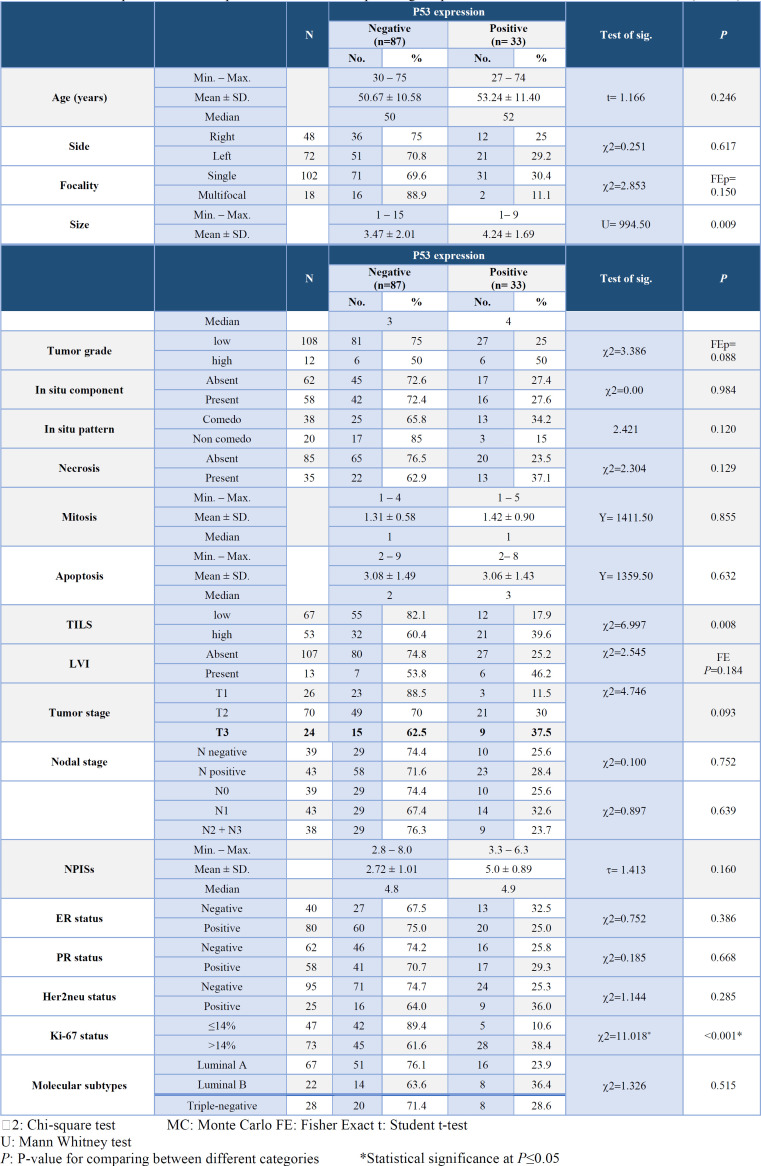

**Survival**

Patients received treatment and follow-up care at the Clinical Oncology and Nuclear Medicine department, Faculty of Medicine, Menoufia University. Survival data collected from patient files were available for 82 cases in the study cohort.

Overall survival time, calculated from the date of diagnosis to the date of death or last follow-up, ranged between 11 and 52 months, with a mean ± SD of 34.93±10.60 and a median of 37 months. Seven cases (8.5%) deceased during the follow-up period, with a median of 37 weeks. Progression-free survival, calculated from the date of diagnosis to the date of progression, ranged between 6 and 52 months, with a mean ± SD of 31.87±13.37 and a median of 35 months.

Twenty-three cases (28%) had a positive family history of breast cancer. Thirteen cases (15.9%) showed metastasis to distant organs. Regarding comorbidities, thirteen cases (15.9%) had hypertension, seven (8.5%) had diabetes mellitus, six (7.3%) had hepatitis C infection, four (4.9%) had cardiac complications, and four (4.9%) had both diabetes mellitus and hypertension. 

Monitoring the response to treatment revealed that 25 cases had gross disease during treatment. Among them, thirteen cases (52%) showed progressive disease (*P*D), nine (36%) showed partial response (*P*R), two (8%) showed stationary disease (SD), and one (4%) showed complete response (CR). For statistical purposes, cases were divided into PD and non-PD (*P*R+SD+CR) groups.

**Association of Treatment Response with the Clinicopathological Parameters, Including Immunohistochemical Markers (n = 25)**

Progressive disease response exhibited a highly significant association with the presence of a low number of TILs (*P*=0.009), advanced tumor stage (T3) (*P*=0.002), and metastasis to distant organs (*P*≤0.001). Additionally, there was a significant association between progressive disease response and larger tumor size (*P* =0.011). Results from Rcc2, Rac1, and p53 immunostaining indicated a non-significant association with the type of patients' response to treatment. 

**Association of the Metastatic Status and the Clinicopathological Parameters Including Immunohistochemical Markers (n= 82)**

Patients with distant metastasis significantly exhibited larger tumor size (*P*=0.003), a higher Nottingham Prognostic Index (NPI) (*P*=0.007), positive Her2neu status (*P*=0.023), and progressive disease with therapy (*P*<0.001). Consequently, they experienced shorter progression-free survival (*P*FS), shorter overall survival (OS) (*P*<0.001), and higher mortality rates (*P*<0.001). Results from RCC2, Rac1, and p53 immunostaining showed a non-significant association with the metastatic status of the studied cases. 

**Association of the Progression-free Survival and the Clinicopathological Parameters Including Immunostaining Results (n= 82)**

**Fig. 6:**
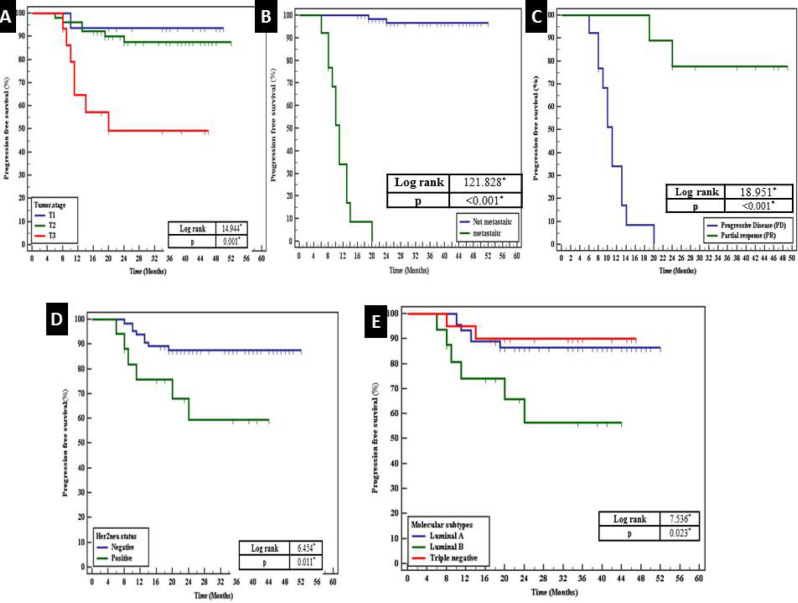
Short progression-free survival is associated with the advanced tumor stage (T3) (*P*=0.001) (A) presence of metastasis to distant organs (*P*<0.001) (B) progressive disease (*P*<0.001) (C) positive Her2neu status (*P*=0.011) (D) Luminal B cases (*P*=0.023) (E)

A highly significant association was observed between the shorter progression-free survival (*P*FS) and advanced tumor stage (T3) (*P*=0.001), the presence of metastasis to distant organs (*P*<0.001), and progressive disease in response to therapy (*P*<0.001). Additionally, shorter PFS exhibited a significant association with positive Her2neu status (*P*=0.011) and the Luminal B molecular subtype (*P*=0.023) (Figure 6). Multivariate analysis revealed that the presence of metastasis to distant organs was the most independent factor affecting PFS (*P*=0.001) (Table 6). Results from RCC2, Rac1, and p53 immunostaining showed a non-significant association with PFS survival.

Survival outcomes by the clinicopathological parameters including immunostaining results (n= 82) 

**Fig. 7:**
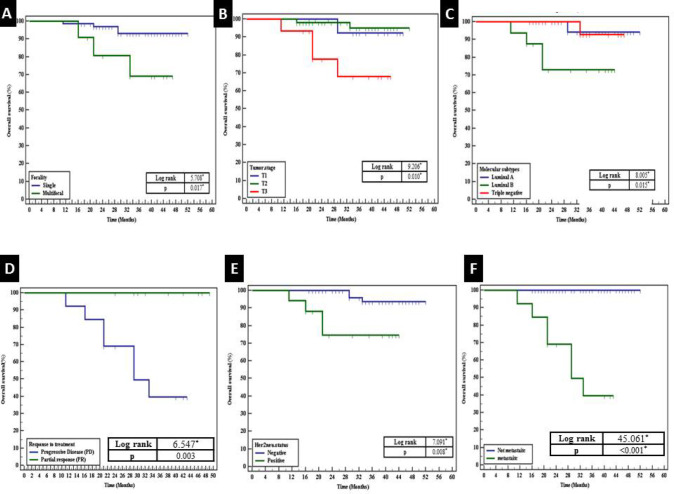
Shorter overall survival in the patients presented by (A) multifocal tumors (*P*=0.017), (B) advanced tumor stage (T3) (*P*=0.010), (C) Luminal B molecular subtype (*P*=0.015), (D) progressive disease response to treatment (*P*=0.003), (E) positive Her2neu status (*P*=0.008) and (F) presence of metastasis to distant organs (*P*<0.001).

Significantly shorter overall survival (OS) was observed in patients presenting with multifocal tumors (*P*=0.017), advanced tumor stage (T3) (*P*=0.010), and the Luminal B molecular subtype (*P*=0.015). Moreover, a highly significant shorter OS was noted in patients with progressive disease response to treatment (*P*=0.003), positive Her2neu status (*P*=0.008), and the presence of metastasis to distant organs (*P*<0.001) (Figure 7). However, Multivariate analysis revealed that none of the aforementioned factors could be considered as independent factors affecting overall survival (Table 7). Results from RCC2, Rac1, and p53 immunostaining showed a non-significant association with overall survival.

**Table 6:** Multivariate COX regression analysis for the parameters affecting progression-free survival

	p	HR (LL – UL 95%C.I)
Tumor stage (T3)	0.203	0.308(0.050 – 1.892)
Her2neu status	0.308	2.571(0.418 – 15.825)
Molecular subtypes(Luminal A+ B)	0.724	0.714(0.110 – 4.631)
Metastatic status to distant organs	0.001^*^	49.866(4.653 – 534.421)
Response to treatment	–	–

**HR: **Hazard ratio **C.I: **Confidence interval

**LL: **Lower limit **UL: **Upper Limit *Statistical significance at *P*≤ 0.05

Results of RCC2, Rac1, and p53 immunostaining showed a non-significant relationship to PFS survival.

**Table 7:** Multivariate COX regression analysis for the parameters affecting overall survival

	p	HR (LL – UL 95% C.I)
Focality	0.172	5.353(0.483 – 59.367)
Tumor stage (T3)	0.843	0.806(0.095 – 6.842)
Her2neu status	0.634	1.871(0.142 – 24.607)1
Luminal B	0.442	0.268(0.009 – 7.733)
Metastatic status to distant organs	0.953	–
Response to treatment	–	–

**HR: **Hazard ratio

**C.I: **Confidence interval **LL: **Lower limit **UL: **Upper Limit

*: Statistically significant at *P*≤ 0.05

## Discussion

Metastasis often leads to unfavorable outcomes and poor prognoses in cancer patients. Molecular markers and adhesive proteins play crucial roles in regulating metastasis through intricate signaling networks within different cell types. Identifying markers that govern metastasis is paramount for understanding and controlling tumor behavior. In colon cancer, Song,* et al.* demonstrated the significance of p53 and RCC2 signaling in inhibiting random cell migration and metastasis (10). They elucidated that p53 suppresses metastasis by inducing RCC2-mediated inactivation of Rac1. In breast cancer, the expression and correlation of p53/RCC2/Rac1 proteins warrant attention.

In our study, RCC2 exhibited positive expression in 116 cases (96.7%), with Strong intensity detected in 52.6% of cases and high expression demonstrated in 47.5%. Chen,* et al.* reported increased RCC2 expression in breast cancer tissue (4). Additionally, Wang,* et al.* documented elevated RCC2 expression in ER-positive breast cancer (26). Elevated RCC2 expression has also been observed in other tumors such as colorectal cancer (29), gastric cancer (30), and lung cancer (31), suggesting an association between RCC2 and cancer, including breast cancer. 

In our cohort, a strong and high RCC2 expression showed a significant association with the clinicopathological parameters of poor prognostic significance, including large tumor size, frequent mitoses, high Ki-67 status, presence of LVI, and positive Her2neu. Chen,* et al.* corroborated these findings in breast cancer, reporting a significant decrease in tumor size upon silencing of RCC2, which also inhibited tumor progression and metastatic potential. They suggested that RCC2 promotes the proliferation and migration of breast cancer cells (4). Additionally, studies on other cancers confirmed a poor prognostic significance of RCC2 expression and demonstrated its role in cell division, cell cycle progression, cell migration, metastasis, and cancer promotion (30, 31). Therefore, RCC2 could play a pivotal role in breast cancer behavior.

Rac1 showed positive expression in all 120 cases (100%) in our study, with 56 cases (46.7%) exhibiting high expression. Previous studies have confirmed Rac1 expression in breast cancer and suggested its involvement in the cancer progression (32, 33).

In our work, high Rac1 expression showed a significant association with poor prognostic parameters such as high Ki-67 status, presence of LVI, and positive Her2neu. Yamaguchi,* et al.* found that Rac1 expression was associated with poor prognostic factors such as a high Ki-67 proliferation index (34). Moreover, significant associations of Rac1 with poor prognostic parameters were demonstrated in other types of cancers including renal cell carcinoma (35), gastric cancer (36), and hepatocellular carcinoma (37). The significant association between high Rac1 expression and hormone receptors including ER status and Her2neu status in our study has been previously investigated, suggesting that a Rac1 inhibitor may be a promising treatment in endocrine-resistant breast cancer (38, 39). Taken together, Rac1 might serve as a promising therapeutic target in breast cancer.

In our study, high RCC2 and Rac1 expression were significantly associated with frequent apoptosis. Previous findings indicated that increased RCC2 led to attenuation of apoptosis through blocking Rac1-initiated apoptosis. However, Rac1 was known to have a complex role in apoptosis—It may function as either a pro-apoptotic or anti-apoptotic protein, depending on the cellular context and inducers of apoptosis. Thus, the association between RCC2 and Rac1 concerning apoptosis depends on more complex factors that ultimately influence the response of tumor cells to drug-induced apoptosis (40).

P53 showed a positive expression in 33 cases (27.5%) in our study, with strong expression detected in 60.6% of positive cases. Since mutant p53 protein tends to accumulate and stabilize in the tumor cell nuclei, its detection by immunohistochemistry is interpreted to indicate the presence of a mutation (41). Despite the confirmed loss of function of p53 in breast carcinogenesis, mutations in the gene occur at a significantly lower frequency than in other common solid tumors. Several studies have detected mutant p53 protein overexpression in breast cancer at similar low percentages (16.3-30%) (42-44).

In our cases, there was a highly significant association between positive p53 expression and adverse prognostic factors such as large tumor size and high Ki-67 status. Additionally, p53 expression percent showed a significant positive linear correlation with frequent apoptosis, and mild p53 intensity showed a significant relation with advanced nodal stage (N2+N3). Previous studies have declared the association of p53 with poor prognostic parameters including high tumor grade, high Ki-67 proliferation index, Her2neu, and triple-negative expression (41, 45-47). However, it was reported that the p53 mutation did not impact the outcome of breast cancer (48). These contradictory results might be explained by the fact that p53 mutant subtypes differ and indicate a debated value of p53 protein overexpression in breast cancer prognosis. Dobes,* et al.* declared that the *TP53* mutation type affected the prognostic significance of breast carcinoma, with nonsense mutations being the most important for determining breast cancer patients' outcomes (49). Thus, the role of the p53 protein in breast cancer is not established, and its clinical application is still debated. 

There was a highly significant direct correlation between each immunohistochemical marker in this study and the other two markers. RCC2 percent positively correlated with Rac1 percent, suggesting that RCC2 might regulate Rac1 activity in breast IDC. Previous studies suggested that RCC2 acts as a Rac1 regulator, promoting mitosis and cell division (50). Inconsistent results regarding the mechanism of regulation were suggested (49, 50). RCC2 binds to the switch region, guanine-nucleotide-exchange factors (GEF), of Rac1 and controls its on/off activity (50). Additionally, RNA interference (RNAi)-mediated depletion of RCC2 promoted the activation of Rac1 (51), suggesting its inhibitory role. Another possible mechanism of regulation of RCC2 and Rac1 was that Coro1C might affect Rac1 localization by interaction with the RCC2–Rac1 complex and release Rac1 from RCC2 for activation. It was also considered that inhibition of Rac1 in RCC2-null cells inhibited cell metastasis (50). 

In this study, a highly significant direct correlation between RCC2 percent and p53 percent was detected. In agreement with these results, Song* et al.* found that RCC2 was transcriptionally activated by P53 through activating the promoter of RCC2 in colon cancer, and P53 deficiency suppressed RCC2 expression (10). So, P53 could regulate RCC2 activity in breast IDC.

There was a significant positive correlation between P53 percent and Rac1 percent in the studied cases. Previous studies had conflicting results suggesting multiple pathways of interaction (10, 52, 53). This was confirmed when Rac1 was independently activated in P53-deficient B- and T-cell lines (53). Thus, the mechanism that accounts for P53-Rac1 interactions is still incompletely understood. In contrast to our results, Song* et al.* study on colorectal cancer found that RCC2 or p53 deficiency triggers Rac1 activation, and they also established that p53 and RCC2 deficiency promotes random cell migration and loss of RCC2 facilitated tumor metastasis in vivo. In addition, they concluded that the RCC2-P53-Rac1 signaling pathway was important for the suppression of tumor metastasis in colon cancer (10). Considering these conflicting results, the RCC2-P53-Rac1 signaling pathway might have a role in breast cancer; however, further investigations are required.

Survival analysis revealed that progressive disease response was significantly associated with the presence of a low number of TILs, larger tumor size, advanced tumor stage, and metastasis to distant organs. In addition, patients with distant metastasis significantly had large tumor size, higher NPI, positive Her2neu status, and progressive disease with therapy. Moreover, patients with shorter PFR and Shorter overall survival significantly experienced more metastasis to distant organs, positive Her2neu status, and Luminal B molecular subtype. Multivariate analysis revealed that the presence of metastasis to distant organs was the most independent factor affecting PFS. These expected prognostic variables were previously confirmed in other studies (54-56).

 In this study, RCC2, Rac1, and P53 showed a non-significant association with overall survival, Progression-free survival, and response to treatment. In contrast to these results, Overexpression of RCC2 has been associated with shorter overall survival, poor prognosis, and recurrence survival rates in breast carcinoma (4) and lung cancer (31). These inconsistent results could be due to different sample sizes and different tumor types. These results might suggest that RCC2 played different functions in different tumors which may need further exploration.

The prognostic significance of Rac1 in breast carcinoma was previously investigated and showed inconsistent results. Takagi* et al.* detected similar results (57) while Yamaguchi *et al*. found a significant correlation between Rac1-GTP status and increased recurrence and breast cancer-specific mortality. In addition, multivariate analyses considered Rac1-GTP as an independent worse prognostic factor for both disease-free and breast cancer-specific survival. Rac1-GTP correlated with a worse prognosis in the patients who had received adjuvant chemotherapy or endocrine therapy (34). Different inconsistent results were detected in other studies (23, 57). This could be explained by the fact that the expression of Rac1 either by immunohistochemistry or protein level did not indicate its activity, and this could be the cause for these inconsistent results.

Regarding p53, contrary to our results, it was found that the presence of mutant p53 was associated with both adverse breast cancer-specific and overall survival. However, it was specific for certain molecular types, such as HER2-enriched and luminal B (58). These disparities could be explained by the fact that the different p53 mutations have different consequences in breast cancer pathogenesis, and thus different impacts on patient outcome and response to therapy could be detected. 

## Conclusion

The high expression of RCC2, Rac1, and P53 in breast cancer might indicate their role in its behavior. The association of RCC2 and Rac1 with poor prognostic clinicopathological factors could help in the prediction of tumor progression, and thus targeted therapy could be helpful in the treatment of breast cancer. The RCC2-P53-Rac1 signaling pathway might have a role in breast cancer; however, further investigations on a larger scale are required.
